# Analysis of the mediating effect of sleep quality between anxiety state and quality of life in patients with functional dyspepsia

**DOI:** 10.3389/fpsyt.2026.1911650

**Published:** 2026-09-16

**Authors:** Xiaoming Huang, Changjuan Lei, Wei Xiao, Heli He

**Affiliations:** 1 Department of Traditional Chinese Medicine, The First Affiliated Hospital of Changsha Medical University, Changsha, Hunan, China; 2 Department of Gastroenterology, The First Affiliated Hospital of Changsha Medical University, Changsha, Hunan, China

**Keywords:** anxiety state, functional dyspepsia, mediating effect, quality of life, sleep quality

## Abstract

**Objective:**

To explore the correlation between anxiety status, sleep quality, and quality of life in patients with functional dyspepsia (FD) and to analyze the mediating role of sleep quality between anxiety status and quality of life.

**Methods:**

A retrospective analysis was performed on 247 patients with FD between January 2023 to December 2025. The assessments included general information, the SAS, PSQI, and SF-36. Using SPSS 26.0 and PROCESS, we built an intermediary model to verify sleep quality as a mediator between anxiety and quality of life in patients with FD.

**Results:**

SAS scores correlated positively with PSQI scores (*r* = 0.543, *P* < 0.05) and inversely with SF-36 scores (*r* = -0.775, *P* < 0.05). The PSQI score was negatively associated with the SF-36 score (*r* = -0.591, *P* < 0.05). Anxiety had a significant total effect on quality of life (*β* = -0.775, *P* < 0.001). After controlling for mediator variables, the direct effect of anxiety on quality of life remained significant (*β* = -0.644, *P*=<0.001). The indirect effects of sleep quality [*β* = -0.131, Boot 95% CI (-0.184, -0.082)] were significant, and 16.88% of the total effect was attributed to this mediating role.

**Conclusion:**

Anxiety not only directly reduces the quality of life of patients with FD, but also indirectly affects their quality of life by worsening sleep quality. Sleep quality is an important mediating variable between anxiety and quality of life.

## 1 Introduction

Functional dyspepsia (FD) is one of the most common functional gastrointestinal diseases in clinical practice, which refers to the typical symptoms of digestive disorders such as postprandial bloating, early satiety, upper abdominal pain, and burning sensation in the upper abdomen that cannot be explained by organic, metabolic, or systemic lesions detected by gastroscopy, imaging, and laboratory examinations (1, 2). According to a research report (3), the overall prevalence of FD worldwide is approximately 8.4%, with a higher incidence in females than in males. The proportion of middle-aged and young people is relatively high, making it a highly prevalent functional digestive system disease worldwide. Although FD has no clear organic pathological damage and no risk of mortality, long-term and persistent physical discomfort symptoms can continue to interfere with the patient’s daily diet, sleep, and social activities, leading to repeated and prolonged illness, long treatment cycles, and seriously affecting the patient’s physical and mental health and social function (4). Anxiety, depression, and other adverse psychological states are critical risk factors for FD, with over 50% of patients with FD experiencing varying degrees of anxiety (5). Long-term anxiety can disrupt the autonomic nervous system function of the body, leading to abnormal gastrointestinal peristalsis and increased visceral hypersensitivity, further exacerbating the symptoms of indigestion (6). Previous studies have shown (7) that anxiety not only exacerbates the severity and frequency of clinical symptoms in patients with FD, but also directly damages their physiological, social, and psychological health dimensions. It is a core psychological risk factor that leads to a decline in the quality of life (QoL) of patients with FD. Sleep is the core physiological process of the body’s self-repair, neural regulation, and maintenance of emotional homeostasis. Sleep disorders are one of the most common accompanying symptoms in patients with FD, manifested as difficulty falling asleep, easy awakening at night, fragmented sleep, early awakening, daytime drowsiness (8). In theory, negative anxiety emotions can disrupt the body’s sleep homeostasis, trigger sleep disorders, and further exacerbate physical discomfort, impair physical and mental states, and QoL, thereby forming a pathway linking anxiety, sleep disturbances, and diminished QoL. At present, there is a lack of systematically integrated analysis and research examining the intrinsic pathways of the three factors both domestically and internationally. Moreover, it remains unclear whether sleep quality mediates the relationship between anxiety and QoL. This study investigated the mediating effect of sleep quality on the association between anxiety and QoL in patients with FD, providing new insights and evidence for the integrated diagnosis and treatment of functional gastrointestinal diseases.

## 2 Data and methods

### 2.1 Research objects

A retrospective analysis was conducted on the clinical data of 247 patients with FD admitted to our hospital between January 2023 and December 2025. The inclusion criteria were as follows: ① met the Rome IV diagnostic criteria for FD (9) and were diagnosed with FD; ② disease duration ≥ 6 months; ③ clear consciousness and able to understand the questionnaire content and complete it independently; and ④ aged 18–75 years. The exclusion criteria were as follows: ① presence of peptic ulcer, gastritis, or organic diseases of the liver, gallbladder, or pancreas; ② history of gastrointestinal surgery; ③ severe organ dysfunction, including cardiac, hepatic, or renal dysfunction; ④ neurological disorders; ⑤ malignant tumors; ⑥ long-term use of anti-anxiety or antidepressant medications, sedatives, or hypnotics before admission; and ⑦ experience of significantly stressful life events within the past three months.

This was a retrospective clinical study, and the sample size was determined by the actual number of cases that met the inclusion and exclusion criteria during the study period, without the need for prospective sample size estimation. A total of 247 eligible patients were enrolled in the study, including patients with FD who sought medical attention within a designated period. To verify the statistical validity of the sample in this study, a *post hoc* efficacy analysis was conducted in accordance with the “Sample Size Estimation Standards for Clinical Epidemiological Studies” (10) and the sample size design criteria for mediation effect models. The significance level was set at α = 0.05 and statistical power (1 − β) was set at 0.80. The sample size was estimated based on data regarding the associations among relevant variables reported in similar studies on the relationship between physical and psychological factors in FD (11). The estimated correlation coefficient between core variables was approximately 0.5. The minimum sample size required for stable model fitting, calculated using G * Power 3.1 statistical software, was 119 cases. From January 2023 to December 2025, 247 eligible patients with FD who met the inclusion/exclusion criteria were included in the study, which far exceeded the theoretical minimum sample size and ensured reliable mediation model fitting and minimized statistical bias.

### 2.2 Research tools

#### 2.2.1 General background inventory

A general information questionnaire was designed to collect patients’ demographic and clinical data, including gender, age, body mass index (BMI), education level, marital status, disease duration, smoking history, alcohol consumption history, and comorbidities.

#### 2.2.2 Self rating anxiety scale

Anxiety symptoms were measured using Zung’s Self-Rating Anxiety Scale (SAS) (12). This 20-item instrument uses a 4-point Likert scale, and standard score is calculated by multiplying the raw total score by 1.25. Scores above or equal to 50 suggest anxiety, and the severity increases with increasing scores. The scale has high reliability and validity, with a Cronbach’s α of 0.872.

#### 2.2.3 Pittsburgh sleep quality index

Referring to the PSQI (13), the sleep quality of patients in the past month was evaluated, including seven dimensions: sleep quality, falling asleep time, sleep time, sleep efficiency, sleep disorders, daytime function, and use of hypnotic drugs. This was a retrospective study aimed to explore the intrinsic correlation between anxiety and native sleep quality in patients with FD, excluding the confounding effects of hypnotic drugs on sleep indicators. Therefore, in the case screening process, cases that had used hypnotic drugs in the past month were excluded from the study. All enrolled patients had history of using hypnotic drugs, and the PSQI hypnotic drug use dimension scores were all 0, with no inter-group differences or statistical significance. Therefore, this study only used the other six dimensions to comprehensively evaluate patients’ sleep quality. Each dimension is scored 0–3 points, with a total score of 0–18 points. Higher scores indicate worse sleep quality. The Cronbach’s alpha coefficient for this scale was 0.895.

#### 2.2.4 Health survey brief form (SF-36)

We adopted the SF-36 questionnaire (14), which contains 36 items. to measure the participants’ QoL. Scores vary between 0 and 100, with higher values corresponding to better QoL. This internationally accepted instrument had good psychometric performance in the present study, with a Cronbach’s α of 0.912.

### 2.3 Data collection methods

This study was a retrospective data analysis, and all data were obtained from our hospital’s medical record information system, gastroenterology patient follow-up records, and standardized in-hospital assessment database. Cases were strictly screened according to the inclusion and exclusion criteria, and archived general clinical data and psychological, sleep, and QoL assessment records were retrieved. All assessments were completed by dedicated medical staff according to standardized procedures during hospitalization or outpatient visits. The assessment process was standardized, and quality control was maintained. A total of 247 patients with FD with complete and valid data were included in this study. Case screening was performed, and the data were complete and available.

### 2.4 Statistical methods

SPSS 26.0 statistical software and the PROCESS plugin (version 3.5) were used for data analysis. Description of Count Data Example (%): Shapiro-Wilk test was used to test the normality of the measurement data. All measurement indicators in this study followed a normal distribution and are described using (). The variance inflation factor (VIF) was applied for multicollinearity testing. No obvious collinearity was observed when VIF < 5. Pearson correlation analysis was used to explore the correlation between variables for measurement data that conform to normal distribution. Model 4 in the PROCESS plugin was used to test the mediating effect, and the Bootstrap method (repeated sampling 5000 times) was used to calculate the 95% confidence interval (CI). If the CI does not include 0, the mediating effect was valid. Statistical significance was set at P < 0.05.

## 3 Results

### 3.1 General information of patients

Among the 247 patients with FD, 142 (57.49%) were male and 105 (42.51%) were female patients. The patients were aged 20–75 years, with a mean age of 49.74 ± 15.81. BMI ranged from 17.94 to 25.10 kg/m², with a mean value of 21.62 ± 2.01 kg/m². Regarding educational level, 23 patients (9.31%) had a primary school education, 91 (36.84%) had a junior high school education, 69 (27.94%) had a high school or vocational school education, and 64 (25.91%) had a college or higher education. Marital status included 21 unmarried patients (8.50%), 204 married patients (82.59%), and 22 divorced or widowed patients (8.91%). The disease duration ranged from 7 to 13 months, with a mean of 9.98 ± 1.95 months. There were 44 smokers (17.81%), 32 drinkers (12.96%), 33 patients with hypertension (13.36%), 47 patients with diabetes (19.03%), and 18 patients with hyperlipidemia (7.29%).2.2 Score of each variable.

Among 247 patients, the mean SAS score was 47.80 ± 6.69, the mean PSQI score was 8.10 ± 0.88, and the mean SF-36 score was 68.45 ± 4.99, as shown in **Table 1**.

**Table 1:** Score of each variable( $\bar{x}$ ± s, score).

Project	Score
SAS Total Score	47.80 ± 6.69
PSQI Total Score	8.10 ± 0.88
Sleep quality	1.88 ± 0.32
Falling asleep time	0.92 ± 0.30
Sleep time	1.14 ± 0.36
Sleep efficiency	1.75 ± 0.43
Sleep disorders	1.11 ± 0.31
Daytime function	1.30 ± 0.36
SF-36 Total Score	68.45 ± 4.99

SAS, Self-Rating Anxiety Scale; PSQI, Pittsburgh Sleep Quality Index; SF-36, Short Form-36 Health Survey.

### 3.2 Correlation analysis between variables

Correlation analysis revealed a positive link between SAS and PSQI (*r* = 0.543, *P* < 0.05), while SAS and PSQI both correlated negatively with SF-36 (*r* = -0.775, *r* = -0.591, *P* < 0.05) (**Table 2**). As all VIF values were less than 5, multicollinearity was not detected, and all variables were eligible for the regression model.

**Table 2:** Correlation analysis of variables.

Variable	SAS total score	PSQI total score	Sleep quality	Falling asleep time	Sleep time	Sleep efficiency	Sleep disorders	Daytime function	SF-36 total score
SAS Total Score	1	–	–	–					
PSQI Total Score	0.289^**^	1	–	–					
Sleep quality	0.234^**^	0.073	1	–					
Falling asleep time	0.137^*^	-0.005	-0.047	1					
Sleep time	0.326^**^	-0.057	0.038	-0.041	1				
Sleep efficiency	0.096	-0.079	-0.037	-0.025	0.050	1			
Sleep disorders	0.185^*^	0.033	-0.085	-0.023	-0.018	-0.078	1		
Daytime function	0.543^**^	0.346^**^	0.327^**^	0.354^**^	0.471^**^	0.281^**^	0.499^**^	1	
SF-36 Total Score	-0.775^**^	-0.224^**^	-0.254^**^	-0.182^*^	-0.245^**^	-0.180^*^	-0.283^**^	-0.591^**^	1

SAS, Self-Rating Anxiety Scale; PSQI, Pittsburgh Sleep Quality Index; SF-36, Short Form-36 Health Survey; 0.185^*^, *P* = 0.003;-0.182^*^, *P* = 0.004;-0.180^*^, *P* = 0.005;^**^P < 0.001.

### 3.3 Correlation analysis

We further analyzed the correlation between BMI and the SAS, PSQI, and SF-36 total scores. BMI was not significantly correlated with the SAS (*r* = 0.040, *P* = 0.533), PSQI (*r* = 0.002, *P* = 0.974), or SF-36 (*r* = 0.039, *P* = 0.545). These results indicate that BMI had no significant independent effect on anxiety, sleep quality, or overall QoL among the enrolled patients with FD, who were mostly of normal weight.

### 3.4 Mediation effect test

A mediation effect test was conducted with anxiety as the independent variable (X), QoL as the dependent variable (Y), and sleep quality as the mediating variable (M). The results showed that anxiety had a significant overall effect on QoL (*β* = -0.775, *P* < 0.001); after controlling for mediator variables, the direct effect of anxiety on QoL remained significant (*β* = -0.644, *P* < 0.001). The indirect effects of sleep quality [*β* = -0.131, Boot 95% CI (-0.184, -0.082)] were significant, with the mediating effect accounting for 16.88% of the total effect, indicating that sleep quality plays a partial mediating role in the relationship between anxiety and QoL (**Table 3**). The mediation effect diagram is shown in **Figure 1**.

**Table 3:** Bootstrap analysis results of the mediating effect of anxiety on quality of life.

Effect	Path relationship	Standardization *β*	*t*	*P*	*SE*	Bootstrap 95%CI		Proportion of effect (%)
						Boot LLCI	Boot ULCI	
Direct effect	SAS→SF-36	-0.644	14.105	<0.001	0.056	-0.547	-0.413	83.12
Mediating effect	SAS→PSQI→SF-36	-0.131			0.048	-0.184	-0.082	16.88
Total effect	SAS→SF-36	-0.775	19.180	<0.001	0.030	-0.637	-0.518	100.00

The significance of the mediating effect is based on Bootstrap 95% CI, where CI excluding 0 indicates significant effect. The direct effect was tested using t-test. SAS, Self-Rating Anxiety Scale; PSQI, Pittsburgh Sleep Quality Index; SF-36, Short Form-36 Health Survey; CI, Confidence Interval; SE, Standard Error.

**Figure 1:**
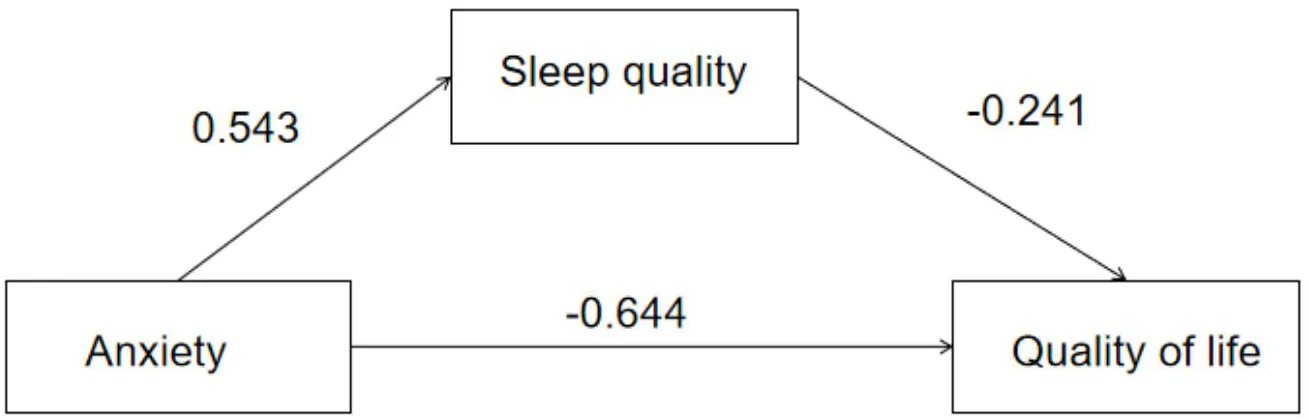
Path diagram of the mediation effect of sleep quality in the anxiety-quality of life relationship.

## 4 Discussion

While prior cross-sectional studies have reported pairwise associations between anxiety, sleep disturbance and quality of life in FD patients, and systematic reviews have described bidirectional links between sleep disturbances and dyspeptic symptoms, this study addresses important research gaps with several novel features. First, unlike most earlier work focusing on gastrointestinal symptom severity or restricted subgroups such as older or pediatric FD patients, we built a single targeted mediation model using the SF-36 total score as the primary outcome in general adult FD patients. We quantified that sleep quality mediated 16.88% of the total effect of anxiety on quality of life, demonstrating both direct and indirect adverse pathways of anxiety toward patient quality of life. Moving beyond pure correlational reporting lacking clinical implications, we translated mediation-effect estimates into practical intervention strategies. Drawing on recent evidence for meditation-assisted therapy, we proposed a multi-dimensional management framework incorporating anxiety screening and standardized sleep interventions to enhance translational value.

### 4.1 The current situation and direct relationship between anxiety, sleep quality, and QoL in patients with FD

The results of this study showed that the total score of SAS in 247 patients with FD was 47.80, indicating that the overall study subjects had significant anxiety, which is consistent with the results of multiple global studies on the psychological status of patients with FD (15, 16). Although FD does not have organic disease support, its symptoms, such as postprandial fullness, early satiety, upper abdominal pain, and burning sensation, persist for a long time, continuously interfering with the patient’s diet, sleep, and daily activities, causing the patient to repeatedly worry about the progression of the disease and the effectiveness of treatment, triggering and exacerbating anxiety (17). Anxiety can disrupt autonomic nervous system function, increase visceral sensitivity, slow gastrointestinal motility, exacerbate digestive symptoms, and form a vicious cycle of symptom anxiety and symptom exacerbation (18). In this study, the PSQI score was 8.10, indicating that patients generally have poor sleep quality, with typical symptoms such as difficulty falling asleep, shallow sleep, easy awakening, early awakening, and decreased daytime function. Sleep disorders are among the most common accompanying symptoms of FD, and their occurrence is influenced by multiple factors. Nocturnal gastrointestinal discomfort symptoms can directly interrupt sleep processes, reduce sleep continuity, and disrupt normal sleep rhythms owing to anxiety-induced mental stress, overthinking, and sympathetic nervous system excitation. Moreover, patients with FD often have autonomic nervous system dysfunction, which can directly affect the regulation of the sleep center and lead to a decline in sleep quality (19). The total score of SF-36 in this study was 68.45, indicating that the overall QoL of patients with FD is at a moderate to low level. Long-term physical discomfort, persistent negative emotions, and sleep disorders combine to weaken the patient’s QoL from multiple dimensions, such as physiological function, psychological state, and social activities, disrupting the normal rhythm of life and social function (20).

According to Pearson’s correlation results, patients with higher anxiety tended to have worse sleep (*r* = 0.543, *P* < 0.05) and poorer QoL (*r* = -0.775, *P* < 0.05). A negative correlation was also found between sleep status and QoL (*r* = -0.591, *P* < 0.05). Collectively, rising anxiety worsens sleep and QoL, and sleep disturbance further reduces QoL. Anxiety, as a core negative psychological factor, not only directly damages QoL, but also disrupts sleep structure and its stability. Lack of sleep and sleep disorders, in turn, exacerbate anxiety and physical symptoms, forming an adverse pathway of mutual influence among the three (21). Billey (22) proposed a significant bidirectional relationship between sleep disorders and FD, with mechanisms involving circadian rhythms, visceral sensitivity, and immune system disorders. Wang et al. (23) found that negative emotions not only directly worsen the condition of FD but also indirectly affect it through a chain-mediated pathway between sleep disorders and physical symptoms. This suggests that clinical treatment should simultaneously focus on psychological support, sleep quality, and improvement of physical symptoms of patients. These results are similar to those of the present study.

### 4.2 Mediating effect of sleep quality between anxiety state and quality of life

Anxiety exerted a total effect of -0.775 and a direct effect of -0.644 on QoL. Its indirect influence on sleep quality was -0.131, accounting for 16.88% of the total effect. The 95% CI did not contain 0, confirming a reliable mediating effect. Anxiety can directly reduce QoL or indirectly have negative effects by deteriorating sleep quality. Sleep quality is a key intermediate variable that connects anxiety and QoL. Under anxiety, sympathetic nervous system hyperactivity occurs, and the secretion of stress hormones, such as cortisol and adrenaline, is disrupted. The cerebral cortex remains in a high arousal state, leading to delayed sleep onset, shallower sleep, increased nighttime awakenings, decreased sleep efficiency, increased PSQI scores, and decreased sleep quality (24). Anxiety can lead to sleep disorders, such as difficulty falling asleep, fragmented sleep, and deep sleep loss. As the core physiological process of body and mind repair, sleep quality decline can further weaken patients’ emotional regulation ability and physical tolerance level, exacerbate fatigue, physical discomfort sensitivity, and psychological exhaustion, and form secondary physical and mental damage mediated by anxiety emotions (25, 26). Previous studies (27) have suggested that the association between sleep quality and health-related QoL is entirely mediated by the ability to perform activities of daily living and negative emotional chains. This study focused on patients with FD to verify the mediating pathway of anxiety affecting QoL through sleep quality, expanding the applicability of this model in the field of functional disorders of the digestive system.

### 4.3 Clinical significance and research limitations

In clinical practice, anxiety screening should be included in the routine assessment of FD, and standardized scales should be used to identify high-anxiety populations in a timely manner and perform psychological and behavioral interventions. Simultaneously, systematic sleep hygiene guidance and non-pharmacological improvement measures should be implemented for those with abnormal sleep quality. A multidimensional integrated management model covering gastrointestinal symptom control, anxiety emotion regulation, and sleep quality optimization should be constructed to achieve synchronous improvement of symptoms, emotions, and sleep through multidisciplinary collaboration and to improve patients’ QoL. Recent clinical studies have proposed non-pharmacological adjuvant interventions to alleviate psychological distress, sleep disturbance, and impaired quality of life in patients with FD. Pathania (28) conducted a randomized pilot trial and confirmed that meditation as an adjuvant therapy effectively improves gastrointestinal QoL among outpatients with FD, providing a low-cost, accessible mind-body intervention strategy for routine FD management. Integrating these evidence-based non-drug therapeutic approaches into daily clinical care will help construct a comprehensive mind-gut management system for functional dyspepsia. However, this study has certain limitations. It was a single-center retrospective cross-sectional study with limited sample representativeness, and the data were all self-reported by patients, which may have led to bias. Potential influencing factors, such as depression and social support, were not included; therefore, the explanatory power of the model needs to be improved. The sex distribution of enrolled patients was unbalanced. Males accounted for 57.49% and females accounted for 42.51%, with a statistically significant higher proportion of male participants. In the future, multi-center, large-sample, prospective cohort studies should be conducted, combined with objective evaluation, to clarify the dynamic causal relationship between anxiety, sleep, and QoL. Simultaneously, research models should be expanded to include more psychological and social variables, and clinical controlled trials should be designed based on this to provide evidence-based support for the FD mind-body co-treatment strategy.

Overall, anxiety impacts QoL of patients with FD through two pathways: a direct negative effect, and an indirect effect mediated by worsening sleep quality. Therefore, sleep quality constitutes a critical mediating factor linking anxiety to QoL. It is imperative that clinical teams prioritize the psychological and sleep health of patients with FD by integrating emotional support and sleep care into routine care. Such comprehensive, multi-pronged interventions can effectively reduce symptom burden, mitigate negative emotions, improve sleep, and promote a better QoL among individuals with FD.

## Data Availability

The original contributions presented in the study are included in the article/supplementary material. Further inquiries can be directed to the corresponding author.
